# Capacitive Micromachined Ultrasonic Transducers (CMUTs) for Underwater Imaging Applications

**DOI:** 10.3390/s150923205

**Published:** 2015-09-15

**Authors:** Jinlong Song, Chenyang Xue, Changde He, Rui Zhang, Linfeng Mu, Juan Cui, Jing Miao, Yuan Liu, Wendong Zhang

**Affiliations:** 1 Key Laboratory of Instrumentation Science & Dynamic Measurement, Ministry of Education, North University of China, Taiyuan 030051, China; E-Mails: jinlong_song@126.com (J.S.); hechangde@nuc.edu.cn (C.H.); fly_zr@126.com (R.Z.); muxiaotengjy@163.com (L.M.); cjj0229@sina.com (J.C.); miaojing_apple@126.com (J.M.); liuy_wlj@163.com (Y.L.); 2 Key Laboratory of Science and Technology on Electronic Test & Measurement, North University of China, Taiyuan 030051, China

**Keywords:** CMUT, *C-V* characteristic curve, sound pressure, bandwidth, ultrasonic imaging

## Abstract

A capacitive micromachined ultrasonic transducer structure for use in underwater imaging is designed, fabricated and tested in this paper. In this structure, a silicon dioxide insulation layer is inserted between the top electrodes and the vibration membrane to prevent ohmic contact. The capacitance-voltage (*C*-*V*) characteristic curve shows that the transducer offers suitable levels of hysteresis and repeatability performance. The −6 dB center frequency is 540 kHz and the transducer has a bandwidth of 840 kHz for a relative bandwidth of 155%. Underwater pressure of 143.43 Pa is achieved 1 m away from the capacitive micromachined ultrasonic transducer under 20 $V_{pp}$ excitation. Two-dimensional underwater ultrasonic imaging, which is able to prove that a rectangular object is present underwater, is achieved. The results presented here indicate that our work will be highly beneficial for the establishment of an underwater ultrasonic imaging system.

## 1. Introduction

Underwater imaging has extensive applications, including underwater observation, differentiation between different objects and recreational underwater activities [1]. Optical visibility in very clear water ranges from about 30 to 60 m, however, the waters within harbors, estuaries and ocean environments are turbid, and this leads to low optical visibility, therefore, the capabilities of underwater optical imaging are limited. Fortunately, acoustic waves can penetrate turbid water and even mud [2].

Ultrasonic waves are a type of sound wave that occurs at frequencies of more than 20 kHz, which is the upper limit of human hearing. Ultrasonic waves offer linear propagation as a result of their small wavelengths, high power (because of the high operating frequency), and strong energy transfer abilities. Ultrasonic devices have actually been used for underwater imaging since World War I and in medicine since the 1930s [3]. They are widely used in a variety of applications, including guiding of interventions in blood vessels and in the heart [4,5], and microelectromechanical systems (MEMS)-based microphones and speakers [6].

Piezoelectric ultrasonic transducers based on lead zirconate titanate (PZT) currently dominate transducer technology. However, low operational efficiency, difficulty in processing two-dimensional arrays and narrow operating bandwidths have been drawbacks when using PZT to meet the demands of modern acoustic transducers [7]. Fortunately, advances in micro-fabrication technology have enabled fabrication of the capacitive micromachined ultrasonic transducer (CMUT), which with its wide bandwidth and design flexibility has emerged as a strong candidate to replace the aging PZT transducer technology [8,9,10]. Additionally, other advantages such as their compatibility with integrated circuit (IC) fabrication technology, array configuration capabilities, good acoustic impedance matching with liquids and high operational efficiency have brought CMUTs one step closer to being the next generation transducer technology [11,12,13] and as a result, underwater acoustic imaging using CMUTs is a technology that requires further study.

In 1994, Khuri-Yakub led his team to create the first CMUT with a sealed cavity, meaning that it could be used underwater [14]. Over the past few decades, CMUTs have attracted increasingly intensive research interest [9,15,16,17,18]. A bandwidth of approximately 40% and pressure of 300 Pa underwater when 1 m away from a CMUT under 100 $V_{pp}$ excitation was achieved at an operating frequency of around 25 kHz using only a single underwater CMUT [19]. Wygant researched the use of transducers working under immersion conditions at around 2.5 MHz for therapeutic applications [20]. Logan fabricated a CMUT with a −6 dB relative bandwidth of 120% [21]. Oralkan has realized 2D imaging using a CMUT with a central frequency at 3 MHz, which only limits the range of action of the transducer [1]. In this work a CMUT with a bandwidth of 155% that requires 20 $V_{pp}$ excitation and has a 540 kHz center frequency for use in underwater imaging has been designed, fabricated and tested.

## 2. Structural Design

The frequency, which is closely related to the radius and the thickness of a vibration, is a vital transducer parameter. Because ultrasonic waves can propagate over greater distances at lower frequencies [22] and because the frequency of the underwater acoustic imaging system is in the 100 kHz–2 MHz range [2], the frequency of a transducer designed for underwater performance is determined to be about 300 kHz. Based on our previous study and the process conditions [23], the radius and the thickness of the membrane are determined to be 90 μm and 2.8 μm, respectively.

**Figure 1:**
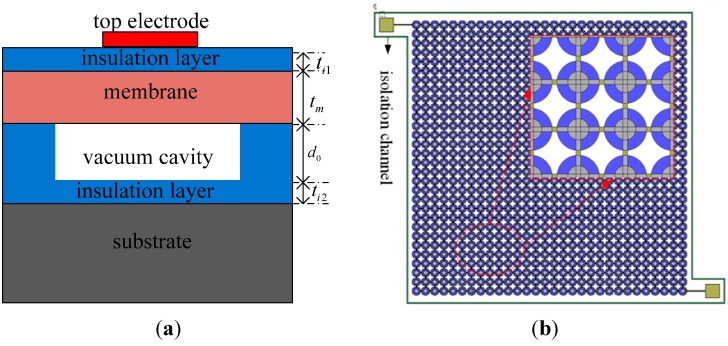
(**a**) The cross-section of a sensitive cell; (**b**) The planform of an element.

A transducer operating in transmitting mode requires a high cavity height to obtain high output pressure, while a transducer working in receiving mode requires only a low cavity height to attain high sensitivity [24]. The optimum cavity height is confirmed to be 0.65 μm after balanced consideration of these requirements. When the size of the top electrode is 40% to 50% of the size of the membrane, the transducer can have a large bandwidth [25]. As a result, the top electrode radius is determined to be 45 μm. A highly-doped silicon wafer is used as the vibration membrane [19]. Unfortunately, this could easily result in ohmic contact between the top electrodes and the membrane, making the membrane become the top electrode. Therefore, a silicon dioxide insulation layer is added between the top electrodes and the membrane. A cross-sectional schematic of a sensitive cell is shown in Figure 1a and the plan form of the element is shown in Figure 1b. The specific parameters of a designed structural element are listed in Table 1.

**Table 1:** The parameters of designed CMUT structure.

Parameters	Value
Membrane radius/μm	90
Membrane thickness/μm	2.8
Electrode radius/μm	45
Electrode thickness/μm	1
Number of cells	900
Electrode insulation layer thickness/μm	0.15
Insulation layer thickness/μm	0.15
Cavity height/μm	0.65

The axial resolution (Δx) is defined as the minimum distance that can be distinguished between two echo sources in the direction of propagation. The axial resolution can be expressed as:

(1) $$\text{Δx}=\frac{n\text{λ}}{2}$$

where *n* is the number of scanning lines, λ is the wavelength and *n*λ is the pulse width.

Increasing the operating frequency and reducing the number of scanning lines can improve the axial resolution of the transducer. For the CMUTs designed in this paper, the axial resolution is approximately 5.55 mm when the number of scanning lines is 3.

The lateral resolution (Δy) is defined as the minimum distance that can be distinguished in the vertical direction of propagation. The lateral resolution can be expressed as:

(2) $$\text{Δy}=\frac{1.22\text{λ}}{\text{D}}\times \text{S}$$

where *D* is the aperture size of the probe and *S* is the distance between the focus and the CMUT.

The lateral resolution at a specific focus can be improved by reducing the operating wavelength and increasing the aperture size of the probe. The probe aperture size for our designed CMUT is 6 mm, and the lateral resolution is approximately 6.78 mm when the distance between the focus and the CMUT is 50 mm.

## 3. Fabrication Process

Like other MEMS transducers, the transducer presented in this paper can be fabricated by the MEMS sacrificial technique [26]. A detailed description of this fabrication process can be found in the literature [8]; however, the required fabrication processes have been summarized briefly in this paper. Before the fabrication process begins, a silicon wafer and a silicon-on-insulator (SOI) wafer should first be prepared. The requirements of the silicon wafer and the SOI wafer are listed in Table 2.

**Table 2:** The detailed parameters of the silicon wafer and SOI wafer.

Parameter	SOI Wafer		Silicon Wafer
Size (inches)	6		6
Conductive type	Device layer	P	P
	Handle wafer	N	
Resistivity (ohm∙cm)	Device layer	0.01–0.08	0.01–0.02
	Handle wafer	0.01–0.02	
Orientation	(100)		(100)
Thickness (μm)	Device layer	2.8 ± 0.1	400 ± 10
	Box layer	0.8 ± 0.08	
	Handle wafer	430 ±15	

**Figure 2:**
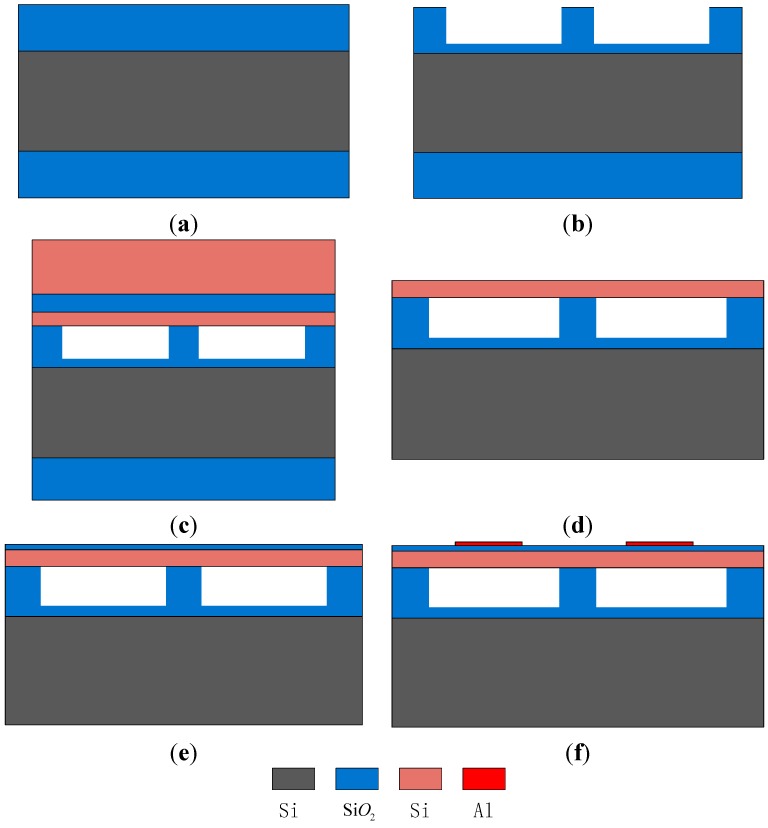
The main fabrication flow-chart.

**Figure 3:**
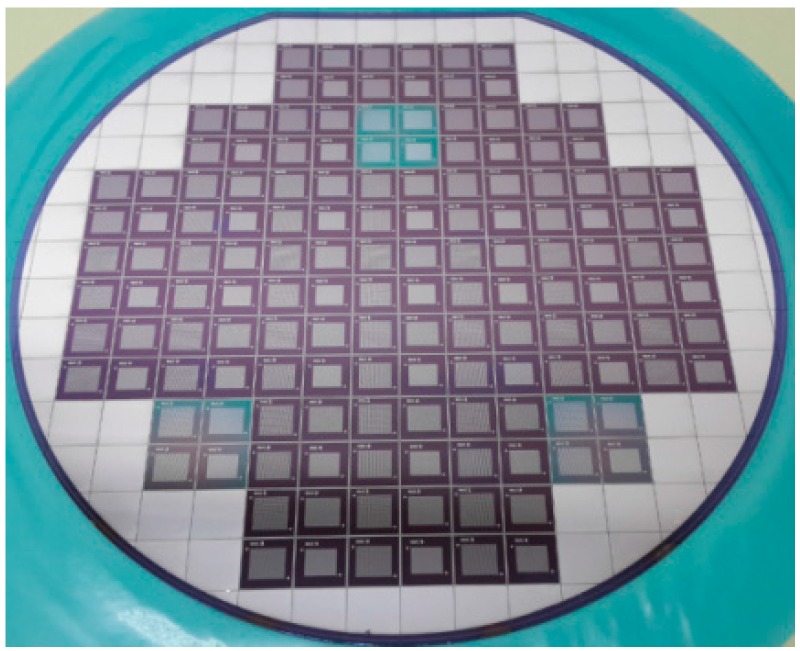
Final fabricated transducers on the 6 inch silicon wafer.

The main fabrication flow-chart is illustrated in Figure 2. The fabricated transducers on a 150 mm wafer are shown in Figure 3.

*Step 1*  Standard RCA cleaning is performed on both the silicon wafer and the SOI wafer to remove organic matter, dust and oxide layers.
*Step 2*  The silicon wafer is then oxidized to form a 0.8-μm oxide layer, which will be part-etched to form cavities, as shown in Figure 2a.
*Step 3*  Gluing and exposure processes are performed on the front area of the silicon wafer; a 0.65-μm oxide layer is etched to form the cavities and a 0.15-μm oxide layer is left behind to prevent membrane contact with the substrate, as shown in Figure 2b.
*Step 4*  The silicon wafer and the SOI wafer are bonded together at low temperature [27,28] and in a vacuum environment [3], as shown in Figure 2c.
*Step 5*  The handle layer of the SOI wafer, the buried oxide (box) layer of the SOI wafer and the oxide layer are etched to produce the basic transducer structure, as shown in Figure 2d.
*Step 6*  A silicon dioxide layer is deposited on the vibration membrane to prevent the formation of an ohmic contact between the top electrodes and the vibration membrane, as shown in Figure 2e.
*Step 7*  A metal layer is sputtered on the vibration membrane by the evaporation method, and the top electrodes and pads are formed by the peeling method, as shown in Figure 2f.

In this process, low temperature wafer bonding technology is vital. Low temperature processing can effectively avoid problems such as introduction of thermal stress, possible contamination, defect generation and doping profile broadening, and also increases the bond strength [27]. Sealed cavities can prevent the hydrolysis of water under high electric fields, thus reducing the energy losses and effectively averting electric breakdown [28].

## 4. Experimental Results

### 4.1. C-V Characteristics

**Figure 4:**
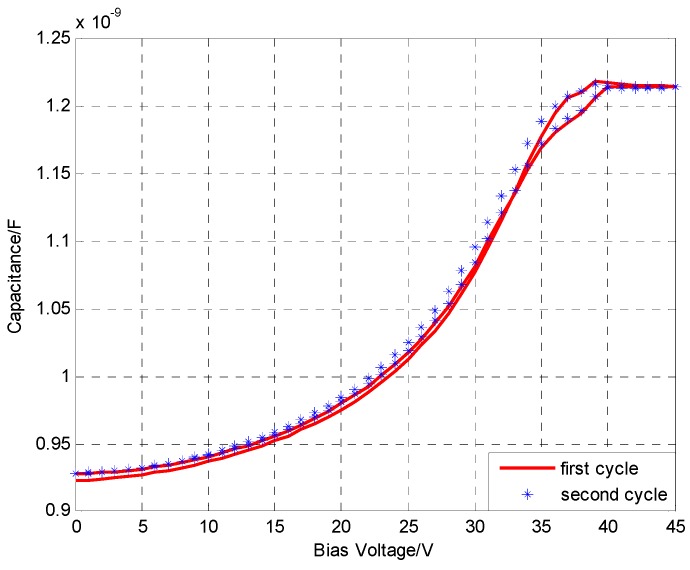
The *C-V* character.

The Agilent 4284A PRECISION LCR METER is used to analyze the effects of the DC bias voltage on the transducer capacitance. The oscillation frequency range of the LCR meter is from 20 Hz to 1 MHz. The DC bias voltage ranges from 0 V to 45 V and then from 45 V back to 0.0 V in a cycle. When the scan cycle number is two, the voltage step length is 0.5 V and the operating frequency is 1 MHz, the *C*-*V* characteristic curve is plotted as shown in Figure 4. The black curve and the green curve represent the first and second cycles, respectively. The red curve and the disperse points represent the average capacitance under different bias voltages.

The capacitance basically remains unchanged when the DC bias voltage ranges from 40 V to 45 V. This phenomenon can be accounted for by the reason that the membrane has collapsed. As a result, the collapsed voltage is 40 V. Additionally, the deviation between the output capacitance values is small when the DC bias voltage is increasing and when the DC bias voltage is decreasing. This indicates that the transducer has a fine hysteresis performance. Moreover, the consistency between the output capacitance values of the first and second cycles is also fine. This implies that the transducer has excellent repeatability performance.

### 4.2. Underwater Experiments

#### 4.2.1. Output Pressure and Bandwidth Testing

Under the hypothesis that ${\text{V}}_{0}\;$ (V) is the collected voltage and *S* (dB) is the sensitivity of the standard hydrophone, the sound pressure level (SPL) can be expressed as:

(3) $$\text{SPL}=20\log_{10}(V_{0})-S$$

The pressure can then be expressed as:

(4) P=10SPL20·Pref

where $P_{ref}=1\text{ μPa}$.

**Figure 5:**
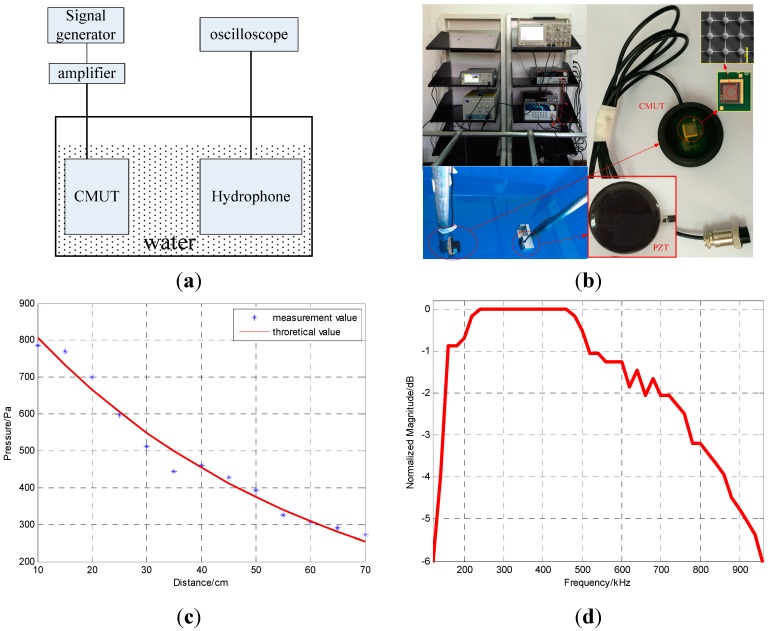
(**a**) The experimental schematic diagram (**b**) The experimental configuration (**c**) the measurement and theoretical pressures at different distances (**d**) The −6 dB bandwidth.

The experimental schematic diagram and configuration are shown in Figure 5a,b, respectively. The left CMUT is driven by multiple cyclical bursts of sinusoids from the signal generator (Agilent 33521A) amplified by a high voltage amplifier (HA-205) at 250 kHz. The bias and excitation voltages are 20 V and 20 $V_{pp}$, respectively. 

**Table 3:** The sound pressure at different distances.

	Distance/cm	Measurement Pressure/Pa	Theoretical Pressure/Pa	Derivation Pressure/Pa
1	10.00	786.7700	807.3977	20.6277
2	15.00	769.6664	733.4918	−36.1746
3	20.00	701.2516	666.3509	−34.9007
4	25.00	598.6294	605.3558	6.7264
5	30.00	513.1109	549.9440	36.8331
6	35.00	444.6961	499.6043	54.9082
7	40.00	461.7998	453.8725	−7.9273
8	45.00	427.5924	412.3269	−15.2655
9	50.00	393.3850	374.5841	−18.8009
10	55.00	324.9702	340.2962	15.3260
11	60.00	307.8665	309.14684	1.28034
12	65.00	290.7628	280.8488	−9.9140
13	70.00	273.6591	255.1410	−18.5181

The −6 dB bandwidth of the hydrophone is 31% and the sensitivity is −199.8 dB @ 250 kHz (0 dB re 1 V/μPa). Based on Equations (3) and (4), the measurement pressures at different distances away from the CMUT are obtained and are plotted in Figure 5c and listed in Table 3.

If the pressure at the sound source is P0, the distance between the sound source and a point on the sound source axis is *x* and the attenuation coefficient is α, the pressure (P) at the point can be expressed as [29]:

(5) $$\text{P}={\text{P}}_{0}e^{-\alpha x}$$

The pressure at the sound source (983.9 Pa) and attenuation coefficient (0.01946) are obtained from the measurement pressure and distance data. Equation (5) can then be written as:

(6) $$\text{P}=978.3e^{-0.0192x}$$

The theoretical and measured sound pressures at various distances are plotted in Figure 5a and listed in Table 3. The average error between the theoretical and measured values is only 4.5%. Additionally, 143.43 Pa pressure is achieved underwater at a distance of 1 m from the CMUT under 20 ${\text{V}}_{\text{pp}}$ excitation. A frequency domain plot of the signal received by the hydrophone is shown in Figure 5b. The −6 dB center frequency is 540 kHz and the transducer has a bandwidth of 840 kHz for a relative bandwidth of 155%. This bandwidth implies that the designed structure achieves the intended aim of increasing the bandwidth.

#### 4.2.2. Distance Testing

**Figure 6:**
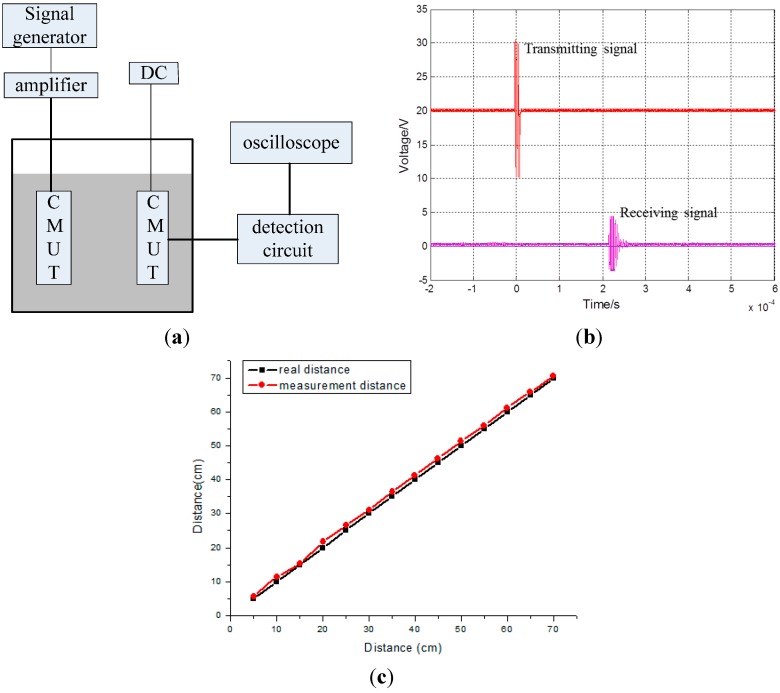
(**a**) Schematic diagram of the distance testing experiment; (**b**) The transmitting and receiving signal when the distance between the two CMUTs; The frequency of the transmitting signal is 250 kHz; (**c**) The measurement and real distances between the two transducers.

The hydrophone is then replaced by a CMUT; an experiment schematic is shown in Figure 6a. When the distance between the two CMUTs is 30 cm, then the filtered signal is as shown in Figure 6a. The first signal is the transmitting signal and the second signal is the receiving signal. 

**Table 4:** The real and measurement distances between the two CMUTs.

	Real Distance/cm	Measurement Distance/cm	Deviation/cm
1	5	5.65	0.65
2	10	11.46	1.46
3	15	15.22	0.22
4	20	21.72	1.72
5	25	26.45	1.45
6	30	31.67	1.67
7	35	36.37	1.37
8	40	41.25	1.25
9	45	46.30	1.3
10	50	51.31	1.31
11	55	55.85	0.85
12	60	61.23	1.23
13	65	65.83	0.83
14	70	70.67	0.67

The measured distance (31.67 cm) between the two CMUTs can be obtained from the speed of ultrasonic propagation underwater (1480 m/s) by multiplying the time that the signal takes to travel from the transmitting CMUT to the receiving CMUT (2.14 × 10^−4^ s). Similarly, measured distances at actual distances of 10–70 cm at 5 cm step increments are plotted in Figure 6b and listed in Table 4.

The results show that the measured distances are greater than the real distances by around 1 cm. The distance between the transducers and the packaging is not considered and the deviation related to the CMUT moving from one location to another can account for this result. In addition, the actual speed of ultrasonic propagation underwater of less than 1480 m/s may also contribute to the result. However, this experiment does demonstrate that the designed CMUT operates well underwater.

#### 4.2.3. Underwater Imaging

**Figure 7:**
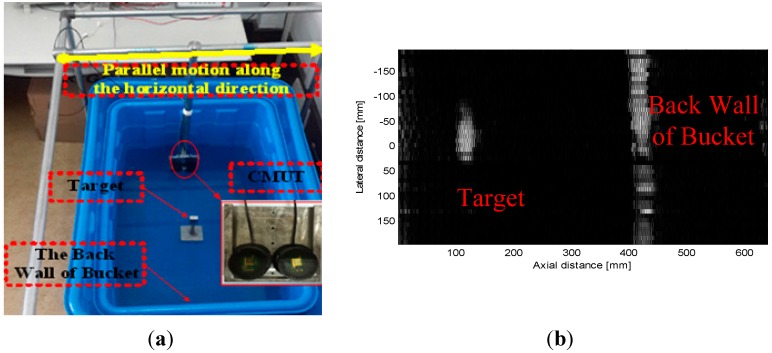
(**a**) The underwater imaging experiment configuration; (**b**) The imaging result of the target.

The experimental underwater imaging configuration is shown in Figure 7a. The imaging target is placed at a specified distance from the transducer. The transducers, of which one is used to transmit ultrasonic waves and the other is used to receive these ultrasonic waves, are moved from the left side to the right side of the imaging target. The initial 2D ultrasonic imaging result is obtained through bandpass filtering, envelope detection, logarithmic compression and image processing, with results as shown in Figure 7b.

While the edge of the target imaging result is fuzzy, we can see that the target is obviously there. Therefore, this experiment has basically demonstrated that the designed transducer can be used for underwater imaging. 1D arrays and 2D arrays will be studied in the future, and a pronounced increase in the imaging results will be obtained.

## 5. Conclusions

A CMUT with an insulation layer appended between its top electrodes and vibration membrane for underwater imaging is designed, fabricated and tested in this paper. The CMUT shows fine hysteresis and repeatability performances. The 155% bandwidth of the transducer proves that the designed structure is beneficial for increasing transducer bandwidth. Distance and imaging experiments demonstrate that the designed transducer shows promise for applications in the underwater field.

## References

[1] Oralkan O., Ergun A.S., Cheng C.H., Johnson J.A., Karaman M., Khuri-Yakub B.T. Underwater acoustic imaging using capacitive micromachined ultrasonic transducer arrays. Proceedings of the OCEANS’02 MTS/IEEE.

[2] Sutton J.L. (1979). Underwater acoustic imaging. IEEE Proc..

[3] Oralkan O., Ergun A.S., Johnson J.A., Karaman M., Demirci U., Kaviani K., Lee T.H., Khuri-Yakub B.T. (2002). Capacitive micromachined ultrasonic transducers: Next-generation arrays for acoustic imaging?. IEEE Trans. Ultrason. Ferroelectr. Freq. Control.

[4] Ito S., Suzuki T., Ito T., Katoh O., Ojio S., Sato H., Ehara M., Suzuki T., Kawase Y., Myoishi M. (2004). Novel Technique Using Intravascular Ultrasound-Guided Guidewire Cross in Coronary Intervention for Uncrossable Chronic Total Occlusions. Circ. J..

[5] Courtney B.K., Munce N.R., Anderson K.J., Thind A.S., Leung G., Radau P.E., Foster F.S., Vitkin I.A., Schwartz R.S., Dick A.J. (2008). Innovations in imaging for chronic total occlusions: A glimpse into the future of angiography’s blind-spot. Eur. Heart J..

[6] Park K.K., Lee H., Kupnik M., Khuri-Yakub B.T. (2011). Fabrication of Capacitive Micromachined Ultrasonic Transducers via Local Oxidation and Direct Wafer Bonding. J. Microelectromech. Syst..

[7] Ladabaum I., Khuri-Yakub B.T., Spoliansky D., Haller M.I. Micromachined ultrasonic transducers (MUTs). Proceedings of the 1995 IEEE Ultrasonics Symposium.

[8] Jeong B., Kim D., Hong S., Chung S., Shin H. (2013). Performance and reliability of new CMUT design with improved efficiency. Sens. Actuators A Phys..

[9] Emadi T.A., Buchanan D.A. (2013). Multiple moving membrane CMUT with enlarged membrane displacement and low pull-down voltage. IEEE Electron Device Lett..

[10] Ergun A.S., Yaralioglu G.G., Khuri-Yakub B.T. (2014). Capacitive micromachined ultrasonic transducers: Theory and technology. Am. Soc. Civ. Eng..

[11] Jin X., Ladabaum I., Khuri-Yakub B.T. (1998). The microfabrication of capacitive ultrasonic transducers. J. Microelectromech. Syst..

[12] Salim M.S., Malek M.F.A., Heng R.B.W., Juni K.M., Sabri N. (2012). Capacitive Micromachined Ultrasonic Transducers: Technology and Application. J. Med. Ultrasound.

[13] Cianci E., Foglietti V., Caliano G., Pappalardo M. (2002). Micromachined capacitive ultrasonic transducers fabricated using silicon on insulator wafers. Microelectron. Eng..

[14] Suo X. (2013). Simulation and Deformation Analysis of CMUT Membrane.

[15] Chen J., Cheng X., Chen C., Li P. (2008). A Capacitive Micromachined Ultrasonic Transducer Array for Minimally Invasive Medical Diagnosis. J. Microelectromech. Syst..

[16] Liu J., Oakley C., Shandas R. (2009). Capacitive micromachined ultrasonic transducers using commercial multi-user MUMPs process: Capability and limitations. Ultrasonics.

[17] Doody B.C., Cheng X., Rich A.C., Lemmerhirt D.F., White R.D. (2011). Modeling and Characterization of CMOS-Fabricated Capacitive Micromachined Ultrasound Transducers. J. Microelectromech. Syst..

[18] Helin P., Czarnecki P., Verbist A., Bryce G., Rottenberg X. Severi S: Poly-SiGe-based CMUT array with high acoustical pressure. Proceedings of the 2012 IEEE 25th International Conference on Micro Electro Mechanical Systems (MEMS).

[19] Olcum S., Oguz K., Senlik M.N., Yamaner F.Y., Bozkurt A., Atalar A., Koymen H. Wafer bonded capacitive micromachined underwater transducers. Proceedings of the 2009 IEEE International Ultrasonics Symposium (IUS).

[20] Jin X., Ladabaum I., Degertekin F., Calmes S., Khuri-Yakub B.T. (1999). Fabrication and characterization of surface micromachined capacitive ultrasonic immersion transducers. J. Microelectromech. Syst..

[21] Wong S.H., Kupnik M., Watkins R.D., Butts-Pauly K., Khuri-Yakub B.T. (2010). Capacitive Micromachined Ultrasonic Transducers for Therapeutic Ultrasound Applications. IEEE Trans. Biom. Eng..

[22] Logan A.S. (2010). The Design, Fabrication and Characterization of Capacitive Micromachined Ultrasonic Transducers for Imaing Applications. Ph.D. Thesis.

[23] Li Y., He C., Zhang J., Zhang H., Song J., Xue C. (2014). Design and analysis of MEMS capacitive ultrasonic transducer. Transducer Microsyst. Technol..

[24] Zhuang X. (2008). Capacitive Micromachined Ultrasonic Transducers with Through-Wafer Interconnects. Ph.D. Thesis.

[25] Bozkurt A., Ladabaum I., Atalar A., Khuri-Yakub B.T. (1999). Theory and analysis of electrode size optimization for capacitive microfabricated ultrasonic transducers. IEEE Trans. Ultrason. Ferroelectr. Freq. Control.

[26] Emadi T.A., Buchanan D.A. (2014). Development of a novel configuration for a MEMS transducer for low bias and high resolution imaging applications. Proc. SPIE.

[27] Tong Q.Y., Cha G., Gafiteanu R., Gosele U. (1994). Low temperature wafer direct bonding. J. Microelectromech. Syst..

[28] Tsuji Y., Kupnik M., Khuri-Yakub B.T. Low temperature process for CMUT fabrication with wafer bonding technique. Proceedings of the Ultrasonics Symposium (IUS).

[29] Feng N. (2006). Ultrasonics Handbook.

